# Label-free visualization of fruit lignification: Raman molecular imaging of loquat lignified cells

**DOI:** 10.1186/s13007-018-0328-1

**Published:** 2018-07-13

**Authors:** Nan Zhu, Di Wu, Kunsong Chen

**Affiliations:** College of Agriculture and Biotechnology, Zhejiang Provincial Key Laboratory of Horticultural Plant Integrative Biology/The State Agriculture Ministry Laboratory of Horticultural Plant Growth/Development and Quality Improvement, Zhejiang University, Zijingang Campus, Hangzhou, 310058 People’s Republic of China

**Keywords:** Fruit, Label-free imaging, Lignification, Raman microspectroscopy, Spectral preprocessing

## Abstract

**Background:**

Flesh lignification, leading to increased fruit firmness, has been reported in several kinds of fruit. Understanding the mechanisms underlying fruit lignification is important to optimize the postharvest storage strategies and reduce the quality deterioration of postharvest fruit. Especially cellular level investigation of lignin deposition in fruits provides novel insight for deciphering the mechanisms underlying fruit lignification. The primary objective of this study was to establish a procedure of using Raman microspectroscopy technique to depict fruit lignification at the cell level.

**Results:**

Lignified cells, a special kind of cells contained high lignin content, were found abundantly scattered in red-fleshed ‘Luoyangqing’ loquat. Whereas these special lignified cells were barely detected in ‘Baisha’ loquat flesh. Dominant Raman bands of lignified cells were found primarily attributed to lignin (1664, 1628, 1603, 1467, and 1272 cm^−1^), cellulose (1383, 1124 and 1098 cm^−1^) and pectin (852 and 1740 cm^−1^). The band intensity correlation analysis indicated the peak at 1335 cm^−1^ assigned to either lignin or cellulose in previous works was related to lignin for the lignified cells. Multi-peaks Gaussian fitting successfully resolved the overlapped fingerprint peaks of lignin in 1550–1700 cm^−1^ into three independent peaks, which were assigned to different functional groups of lignin. Furthermore, the spatially resolved Raman images of lignified cells were generated, indicating that lignin and cellulose saturated the whole lignified cells, pectin mainly located in the cell corner, and the parenchyma cells contained little lignin. In addition, both phloroglucinol-HCl staining and autofluorescence analysis confirmed the results of lignin distribution of Raman microscopic analysis.

**Conclusions:**

A procedure for the simultaneous visualization of the main components of the flesh cells without labeling by high-resolution Raman microspectroscopy has been established. With Raman microscopic imaging technique, we can add a microscopic level to cell compositions, essential for a detailed molecular understanding of loquat lignification. Such method can be further used to chemically monitor the textural changes during the ripening process or postharvest storage of other fruits and vegetables.

**Electronic supplementary material:**

The online version of this article (10.1186/s13007-018-0328-1) contains supplementary material, which is available to authorized users.

## Background

Loquat [*Eriobotrya japonica* Lindl.] belonging to the Rosaceae Eriobotrya is an evergreen woody tree native to subtropical China. Currently, loquat is widely cultivated in Korea, Japan, Brazil and Italy [1, 2]. Loquat fruit has abundant triterpenic acids [3], fatty acids, minerals, amino acids, vitamins, soluble sugars [4], phenolics and carotenoids [5]; thus loquat fruit has good antioxidant activities [6, 7] and other pharmacological benefits [8]. In addition to being consumed fresh, loquat fruits are also used for producing jam, jellies, juice, wine, syrup, nectar or as candied food [2, 4].

Loquat fruit is impressionable to nutritional losses, mechanical damage, and microbial decay, making its postharvest period very short [9]. Low temperature storage is widely used to extend the postharvest life of loquat fruit [9, 10]. However, red-fleshed loquat fruit suffers chilling injury when it is stored below 1 °C, depending upon varieties [11]. Studies show that the chilling injury of red-fleshed loquat fruit causes substantial lignification of flesh during postharvest cold storages [10, 12]. In contrast to red-fleshed fruit, white-fleshed cultivar loquat will not suffer lignification during postharvest [13, 14]. The lignification can significantly influence fruit texture, affect the storability and quality of fruits, and eventually reduce consumer acceptance [15].

Understanding the mechanisms underlying fruit lignification is important to optimize the postharvest storage strategies and reduce the quality deterioration of postharvest fruit. Recent physicochemical and molecule biological studies provided insights into the mechanisms underlying loquat lignification [10, 16–20]. The results show that the increment of lignin in loquat fruit is a major factor to its lignification, resulting in high compression resistance and rigidity to the cell walls [10]. It was revealed an increment of firmness for ‘Luoyangqing’ loquat fruits during postharvest storage and the correlation between the lignin and firmness was positive (r = 0.95**) [11]. Research efforts also have been focused on the expression patterns and transcriptional regulation of lignin biosynthesis related genes during the lignification of loquat fruit [14, 16, 17, 21]. However, these physicochemical and genetic studies are based on tissue homogenate, which only obtained the general physicochemical and genetic information of the flesh components at the tissue level and did not provide the insight into the lignification mechanism at the cell level, let alone visualizing the dynamic variations of cell-level distribution of cell wall substances during lignification. To the best of our knowledge, there is few works for a direct study on the mechanism of fruit lignification at the cell level, which is very important for understanding the mechanisms undying lignification in loquat fruit and other fruits.

Light microscopy (LM) is a common used technique to investigate the microstructure of biological tissues and cells. LM provides the view under visible light, therefore is commonly used in tandem with histochemical staining techniques to identify or localize specific substances of biological tissues and cells [22–24]. Mäule and Wiesner reaction were performed to determine the lignin distributions in the cell wall of *Caragana Korshinskii* [25]. Stained by borax methylene blue, the distribution of hesperidin crystals in peel cells of satsuma mandarin was observed by LM [24]. However, the staining procedures might alter the physical properties of samples in an unknown way [26] and dyes used to generate image contrast can modify native cell behavior [27]. Besides, some substances are resistant to being stained by dyes, such as some carbohydrates, causing the difficulty to obtain the distinct images of these substances [26]. Fluorescence techniques based on fluorescent labels or autofluorescence are also used for visualization of intracellular molecules. However, fluorescence labelling is commonly invasive and fluorophore molecules will suffer from a decrease in fluorescence efficiency (photobleaching) during long time imaging [28–30]. Though autofluorescence imaging of lignin is non-invasive and permits the study of the localization of lignin, it cannot provide detailed chemistry structural and compositional information of the lignified tissue. Some other microscopic methods, such as cryo-scanning electron microscopy [31], X-ray imaging [32], immunocytochemistry [33, 34], have also been used for the evaluation of the structural or compositional features of plant cell walls. These methods are selective, expensive and laborious. On the other hand, analytical chemistry methods such as spectrophotometer and high performance liquid chromatography were used to analyze the molecular features of cell walls [10, 35, 36]. However, analytical chemistry works required the extraction of cell wall substances, leading to a destruction of the cells; therefore also cannot provide the spatial distributions of the molecules and functional groups at the cell level, which are related to cell development, cell fate decision-making, evolution and adaptation [37].

Recently, label-free molecular imaging techniques have been utilized for in situ analysis of molecular information from a single cell [38–40]. In contrast to conventional microscopic techniques, label-free molecular imaging techniques can provide comprehensive information about the morphology and molecular structure of target areas at the cell level without staining or labeling samples. Therefore, the information concerning the molecular structure and composition, location as well as dynamic changes over time can be obtained simultaneously at the micro-level in the native cells [41]. The vibrational microscopies based on the Raman scattering can measure frequency information of various chemical bonds, therefore have the capability of providing label-free contrast [38]. Raman spectroscopy based on “Raman effect” was described for the first time in 1928 [42]. It uses a laser light source to generate inelastic scattering. Raman scattering is attributed to the polarizability changes of functional groups caused by molecular vibration (e.g., bond stretching, rotation, torsion) [43]. Raman spectroscopy allows acquiring molecular structural and compositional information of individual features of samples. By combining microscopy with Raman spectroscopy, Raman microscope scans over a sample and measures the Raman spectra of all pixels within the sample at the same time; therefore can provide the spatial distributions and chemical structures of multiple components of samples simultaneously at the microscopic level [44]. In addition, compared with Infrared microscopy, which is another typical label-free molecular imaging technique, Raman microscopy has the advantage of a higher spatial resolution due to the shorter excitation wavelengths and the feasibility of acquiring the spectra on aqueous [38, 45]. In recent years, Raman microspectroscopy has been applied to probe the compositions and structures of plant cell walls at the micro-scale, mainly about woody materials and energy crops, giving new insights into their native cell walls [45–47], as well as genetically modified [48] and pre-processed cell walls [49–52]. The major cell wall polymers, such as pectin [44, 53], hemicellulose [49], cellulose and lignin [45, 46] were visualized in situ by confocal Raman microspectroscopy. In addition, only a few works have been conducted on fruit analysis using Raman microspectroscopy. Spatial distributions of polysaccharides in the tomato cell wall were presented [44] and the variation of the composition of cell wall in apple flesh during both ripening and postharvest storage was investigated [53]. However, these works are mainly focused on fruit softening. To the best of our knowledge, Raman microspectroscopy has not been used to study fruit lignification.

The primary objective of this study was to establish a procedure of using Raman microspectroscopy technique to depict lignification of loquat fruit at the cell level. The main goals of this study were to: (1) observe and compare the lignin distribution of two cultivars of loquat fruit at the macroscopic level; (2) use the laser scanning confocal microscopy (LSCM) with phloroglucinol-HCl staining and LM with autofluorescence for an assessment of the gross localization of lignin in loquat flesh at the cell level; (3) collect the Raman hyperspectral images of a special kind of cells contained a large amount of lignin, called lignified cells, and assign the collected Raman peaks based on the reference Raman spectra of cell wall polysaccharide standards; (4) confirm the assignment of dubious Raman peaks by calculating band intensity correlation and resolve the overlapped Raman peaks; (5) visualize the spatial distributions of lignin, cellulose and pectin in the lignified cells simultaneously; and (6) generate the distribution maps of the functional groups of lignin in the lignified cells.

## Results

### Lignin distribution in the loquat fruit flesh at the macroscopic-scale

**Fig. 1:**
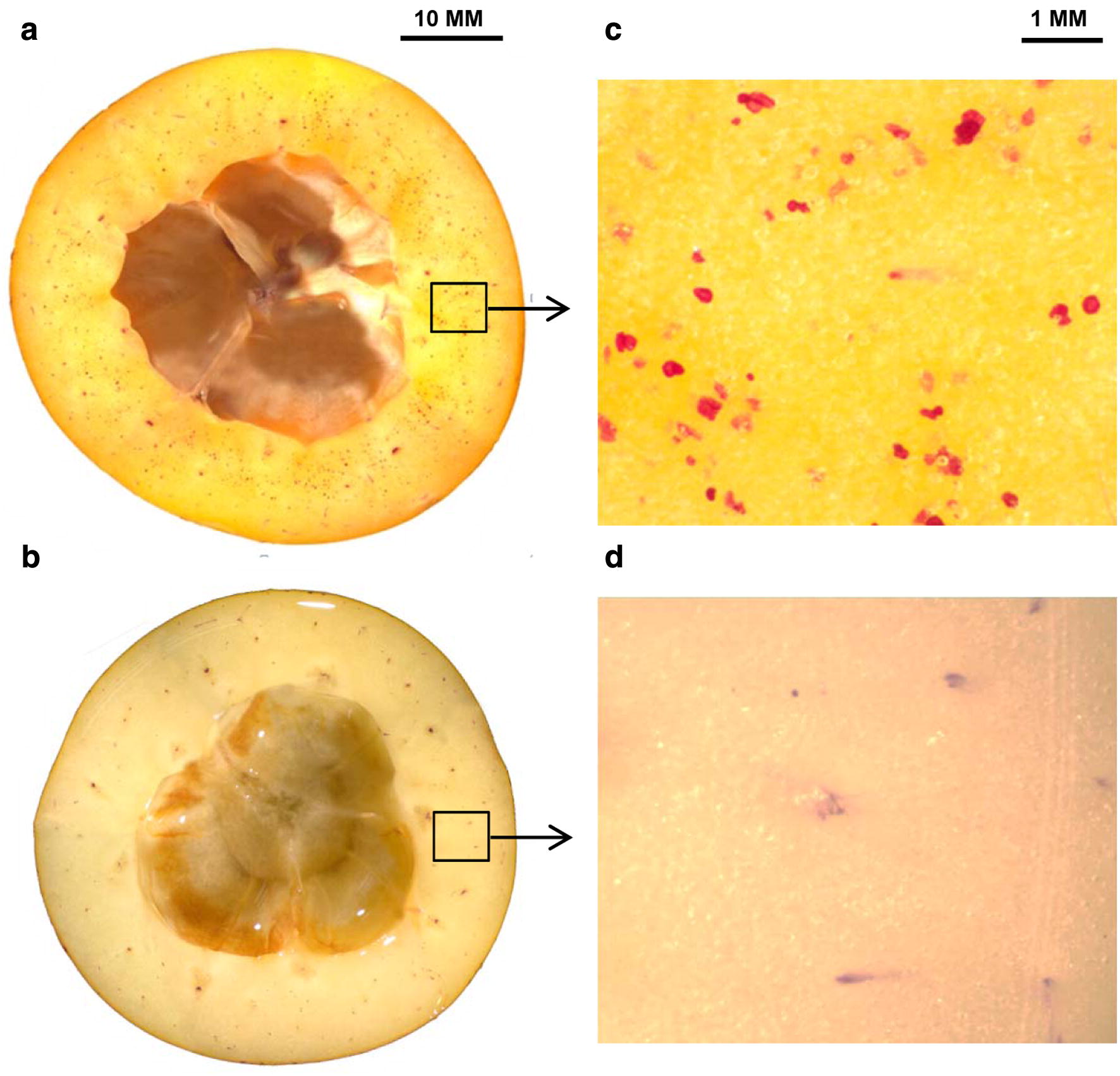
Lignin distribution in red-fleshed LYQ and white-fleshed BS loquat at the macroscopic-scale. **a**, **b** Equatorial plane of LYQ and BS loquat fruit flesh stained with phloroglucinol–HCl. **c**, **d** Enlarged views of the regions in the square boxes of the stained flesh

Currently, investigating biological samples generally has two scale directions. One is imaging a higher spatial resolution at microscopic or even ultramicroscopic scales. The other one is investigating large samples at the macroscopic scale. To study the overall lignification of loquat fruit, the lignin distribution across the flesh of both varieties (red-fleshed and white-fleshed) were studied at the macroscopic-scale using a stereomicroscope in tandem with lignin-staining dye phloroglucinol/HCl (Wiesner reagent). Based on the Wiesner reaction, lignified structures deserve being stained pink-red [54, 55]. As shown in Fig. 1a, c, many spots of flesh were highlighted as pink-red and scattered in the flesh of chilling-sensitive LYQ loquat. These stained flesh spots were lignin deposits. However, in chilling-insensitive BS loquat, such lignin deposits were barely detected (Fig. 1b, d). Macroscopic scale investigation of lignin distribution in loquat fruit not only showed varietal difference but also implied some special lignified structures were existed in chilling-sensitive LYQ loquat.

### Lignin staining and autofluorescence analysis for loquat flesh cells at the microscopic-scale

**Fig. 2:**
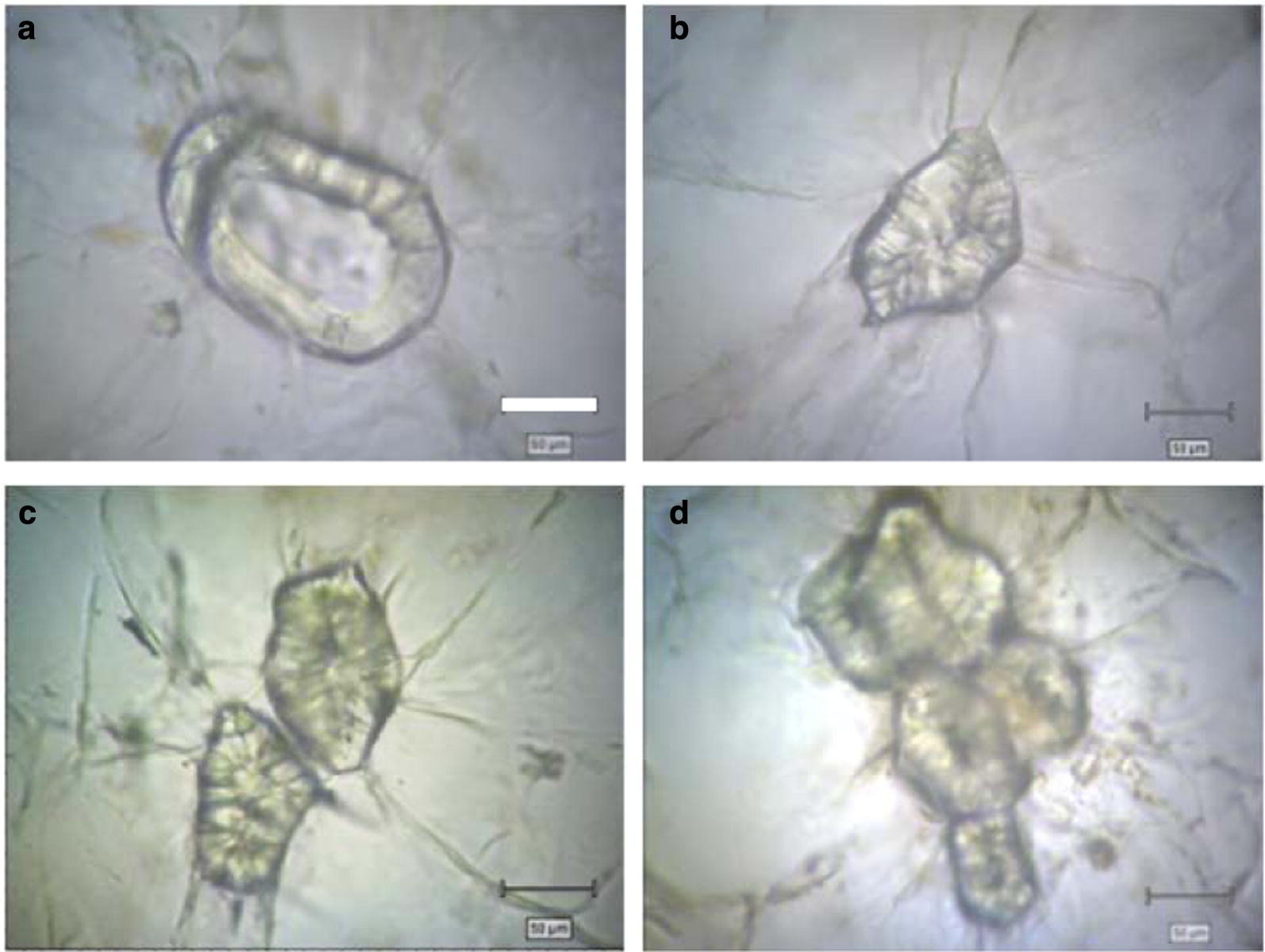
Microscopic images of flesh cells of LYQ loquat fruit. Sclerenchyma cells were found among parenchymal cells in loquat flesh, varying in shapes and sizes. **a** Sclerenchyma cell with highly thickened wall; **b** single solid sclerenchyma cell; **c** double solid sclerenchyma cells; **d** cluster. Scale bar = 50 μm

To gain an insight into the lignified loquat flesh at the microscopic-scale, several microscopic methods were used. First, bright field microscopic images of flesh cells of LYQ loquat fruit were acquired using the Renishaw inVia Reflex Raman microscope. Under the light microscopic view, some sclerenchyma cells were found among parenchymal cells in loquat flesh, varying in shapes and sizes (Fig. 2). The walls of the sclerenchyma cells were obviously highly thickened (Fig. 2a), some even filled almost all the cell volumes (Fig. 2b). Sclerenchyma cell clusters were also found scattered in the parenchyma (Fig. 2c, d).

**Fig. 3:**
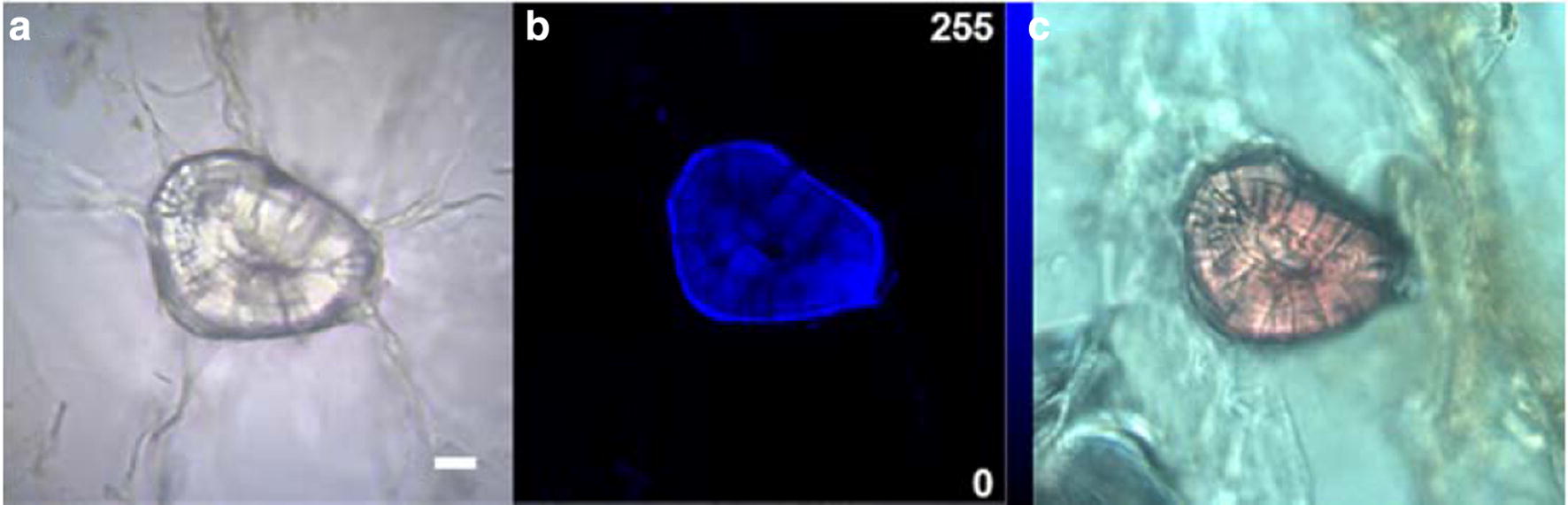
Microscopic images of loquat flesh cells. **a** Bright field images of the flesh cells; **b** Fluorescence images of lignin distribution in the flesh cells strong blue autofluorescence signals were found present in the sclerenchyma cells whereas the parenchyma cells were observed with scarce autofluorescence signals; **c** Sections stained with phloroglucinol–HCl, sclerenchyma cells were observed stained pink-red. Scale bar = 20 μm

Further, the localization of lignin in lignified tissues can be traced by measuring the autofluorescence intensity of the sample excited by UV and visible light [56]. As shown in Fig. 3b, strong blue autofluorescence signals were present in the sclerenchyma cells whereas the parenchyma cells were observed with scarce autofluorescence signals. This indicates that high lignin content exists in the sclerenchyma cells and little lignin deposits in the parenchyma cells. Moreover, the outer boundary of the sclerenchyma cells was detected having stronger autofluorescence signals, indicating that there were higher lignin contents in these regions than the other regions of the sclerenchyma cells. In addition, the Wiesner reaction was conducted on the flesh sections. The microscopic image of the stained sclerenchyma cell was shown in Fig. 3c. The sclerenchyma cell was stained pink-red, indicating that the sclerenchyma cells contained high lignin contents. The parenchyma cells were barely stained (Additional file 1: Figure S1, C, F). Staining of the lignin was consistent with the lignin-autofluorescence results. The other two sample duplicates are shown in Additional file 1: Figure S1. The staining and autofluorescence results indicate that there are a kind of cells containing high lignin content in loquat flesh. We designate these sclerenchyma cells as “lignified cells”.

### Raman spectra of the lignified cells and the reference materials

**Fig. 4:**
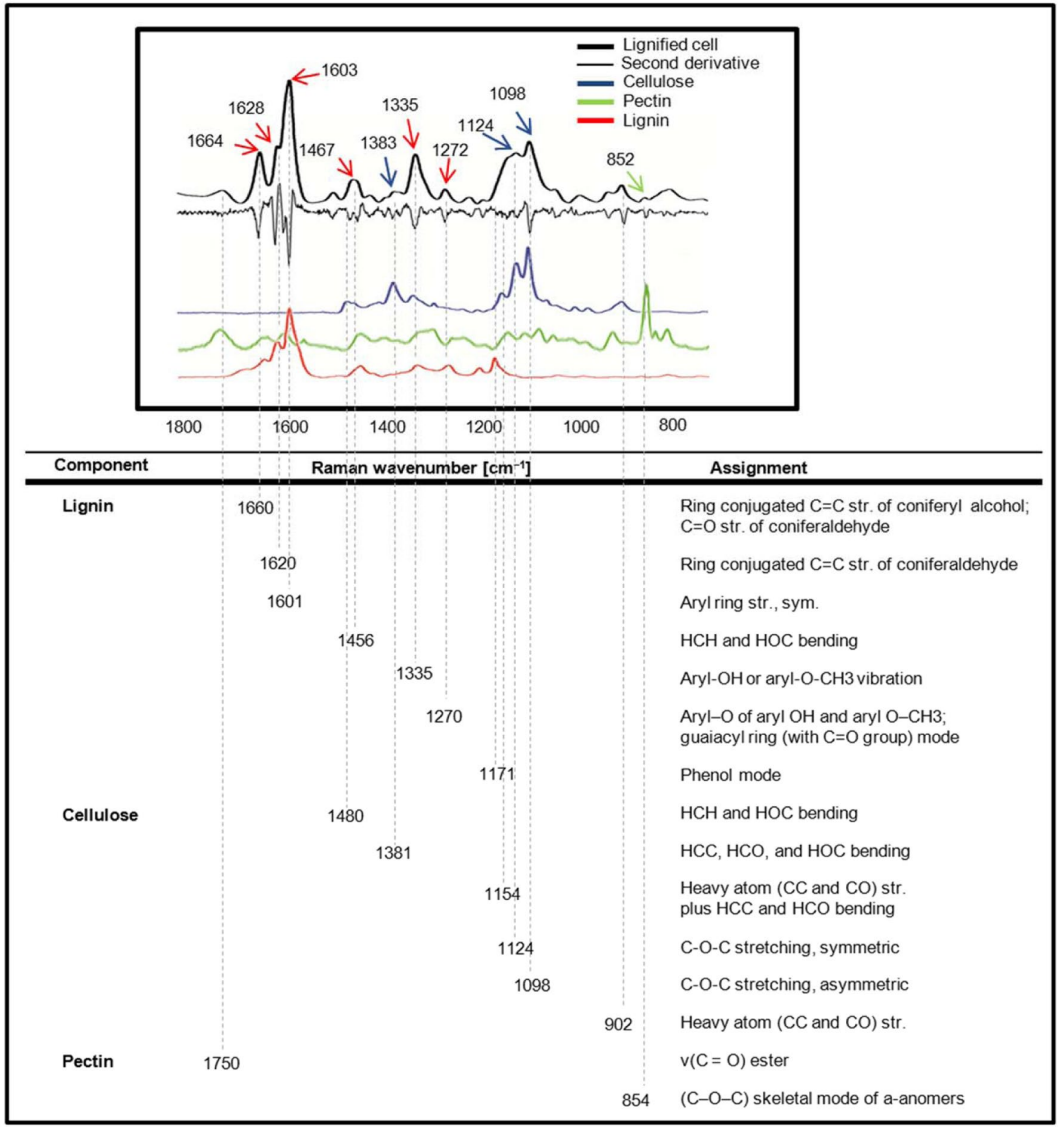
Average Raman spectrum of the lignified cell and its second derivative, the spectra of cell wall polysaccharide standards and their Raman band assignments

An averaged Raman spectrum of a lignified cell was obtained based on its Raman microscopic hyperspectral image data. There were some characteristic peaks in the Raman spectrum (black line in Fig. 4). The second derivative of the Raman spectrum (thin black line in Fig. 4) was calculated to identify the dominant Raman bands of lignified cells. The calculation of minima second spectral derivatization (derivative) reflects the bands’ maxima, therefore it can not only identify small band vibrations, but also determine their positions. The differentiation enhances the number of spectral features and identifies some peaks hidden under broad peaks. In addition, the derivatization process can eliminate constant factors from the spectrum and can reduce some baseline effects. Based on the result of second derivative calculation of the spectrum, dominant Raman bands of lignified cells were identified and are shown in Fig. 4.

**Fig. 5:**
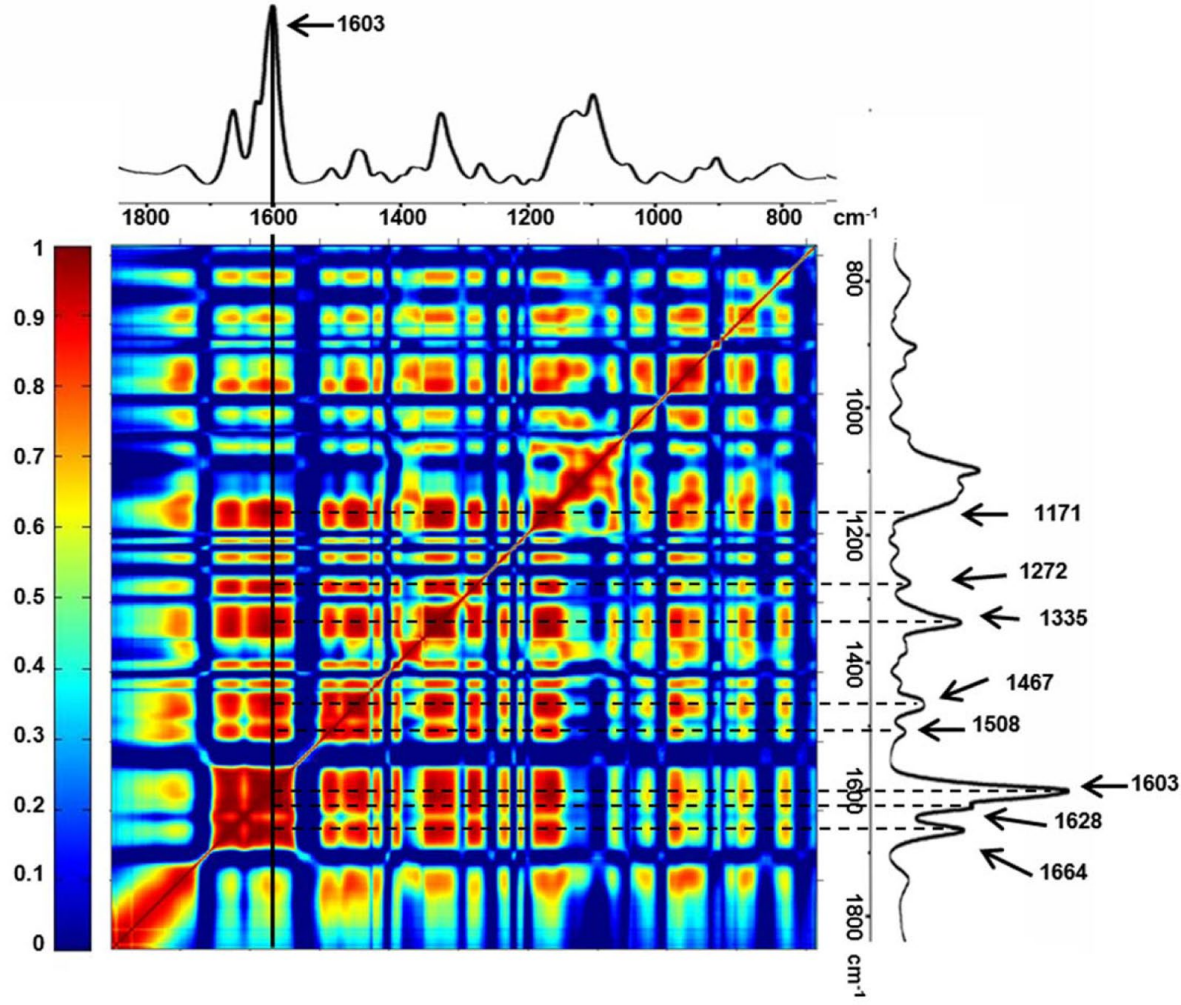
Correlations between spectral bands over the full Raman spectra of the lignified cell in loquat flesh. In the Raman spectra of the lignified cell, bands centered at 1171, 1275, 1335, 1468, 1508, 1627, 1663 cm^−1^ were well correlated with 1603 cm^−1^. 1335 cm^−1^ Raman peak was assigned to lignin in the spectra of the lignified cell

Raman spectra of the lignified cell were comprehensive, carrying a wealth of structural and molecular information of multiple components; therefore the band assignment was carried out based on the Raman spectra of main cell wall polysaccharides standards to better interpret the Raman spectra of the main polysaccharides in the lignified cells of loquat fruit. The reference Raman spectra of the main cell wall polysaccharides, i.e., lignin, cellulose and pectin were measured and are shown in Fig. 4. For better comparison, reference spectra were pre-processed by Min/Max normalization. In the Raman spectrum of lignin standard, spectral peaks were found centered at 1660, 1620, 1601, 1456, 1335, 1270 and 1171 cm^−1^. On the other hand, the spectrum of the cellulose standard had the peaks at around 1480, 1381, 1154, 1124 and 1098 cm^−1^. In addition, the most prominent Raman marker band is centered at 854 cm^−1^ for the pectin standard. Based on the band assignments of the lignin, cellulose and pectin standards (summarized in Fig. 4), the dominant Raman bands of the lignified cells were accordingly attributed to lignin (1664, 1628, 1603, 1467, 1335 and 1272 cm^−1^), cellulose (1383, 1124 and 1098 cm^−1^) and pectin (852 cm^−1^). In specific, the most pronounced peak centered at 1603 cm^−1^, which is the main marker band of lignin within plant cell wall, was assigned to the aromatic C=C vibration. Two shoulder bands at 1664 and 1628 cm^−1^ were also detected. The 1664 cm^−1^ Raman peak was assigned to the conjugated C=C and C=O modes of coniferyl alcohol and coniferaldehyde, respectively, whereas the weak shoulder at 1628 cm^−1^ attributes to only conjugated C=C stretch of coniferaldehyde [49, 57, 58]. As shown in Fig. 4, both lignin and cellulose standards had Raman peaks in the region of 1330–1340 cm^−1^, which made the assignment of Raman peaks of the lignified cell in this region ambiguous. Previous studies also hold different conclusion on the assignment of Raman peaks in this region. Some studies assigned the peaks in the 1330–1340 cm^−1^ region to bond bending of cellulose [45, 49] and, whereas some other researchers [59, 60] attributed the Raman peaks in the 1330–1340 cm^−1^ region to lignin. To clarify the assignments of these dubious Raman peaks of the lignified cells, we used a method based on correlation analysis of intensities of each Raman peak. Because each full Raman spectrum is a molecular ‘fingerprint’ of the sample, Raman peaks of the same individual component exhibit a good correlation. Figure 5 shows the band correlations over the full spectra of the lignified cells. In the Raman spectra of the lignified cell, bands centered at 1171, 1272, 1335, 1467, 1508, 1628, 1664 cm^−1^ were well correlated with 1603 cm^−1^, which is the most prominent and marked Raman peak of lignin. For attributing the band at 1335 cm^−1^, the correlation coefficients between the Raman band at 1335 cm^−1^ and the Raman bands of lignin were calculated. The Raman band centered at 1335 cm^−1^ exhibited high correlation coefficients of 0.889, 0.923, 0.948, 0.902, 0.952, 0.916, and 0.946 with lignin bands at 1171, 1272, 1467, 1508, 1603, 1628, 1664 cm^−1^, respectively. We also calculated the correlation coefficients between the Raman band at 1335 cm^−1^ and the Raman bands of cellulose (1383 and 1098 cm^−1^), which were 0.461 and 0.451, respectively. The correlation coefficients between the signal of the Raman band at 1335 cm^−1^ and the signal of Raman bands of cellulose were low. Therefore, the 1335 cm^−1^ Raman peak for the lignified cells in loquat was mainly attributed to lignin. In addition to lignin, characteristic Raman bands of cellulose at 1124 and 1098 cm^−1^ were observed in the Raman spectra of the lignified cells in loquat flesh. They were assigned to the symmetric and asymmetric stretching vibrations of C–O–C linkages of cellulose, respectively [47, 53, 57, 61, 62]. Because both cellulose and hemicellulose have the main backbone consists of β 1 → 4-linked glucose residues, hemicellulose also contributes to band 1124 and 1098 cm^−1^ [53, 62]. Nevertheless, it should be noted that the two band heights are highly sensitive to the orientation of the cellulose molecule [45, 63]. On the other hand, the band centered at 1383 cm^−1^ was attributed to HCC, HCO and HOC bending of the cellulose and it shows non-orientation-sensitivity [44, 53, 64]. As a conclusion, the assignment of the Raman spectral bands confirmed the presence of lignin in the lignified cells of loquat fruit at the cell level in the native state. Moreover, besides lignin, the lignified cells also contained cellulose.

### Visualization of the distribution of lignin, cellulose and pectin in the lignified cells of loquat fruits

**Fig. 6:**
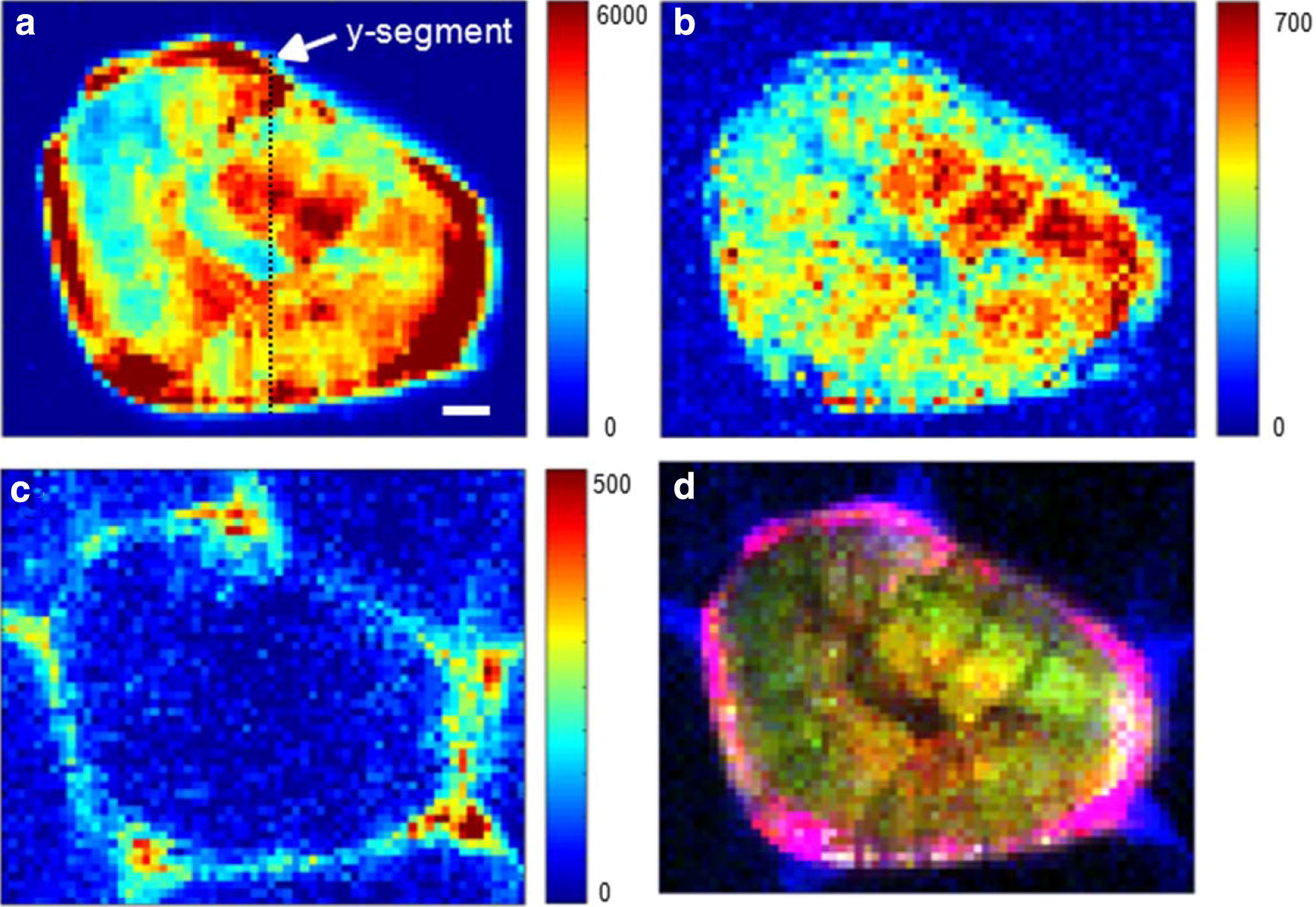
Spatial distribution of cell wall polysaccharides of loquat flesh generated based on the Raman hyperspectral images. **a**–**c** Distribution of lignin, cellulose and pectin by integrating over the wavenumber region from 1550 to 1700, 1361 to 1395 and 843 to 872 cm^−1^, respectively. **d** An merged pseudo-color image showing the spatial distribution of the cell wall polysaccharides simultaneously. Scale bar = 20 μm

In general, a Raman hyperspectral image contains hundreds or thousands of spectra. Each of them carries a wealth of not only chemical but also position-resolved information. Raman imaging allows the simultaneous generation of several two-dimensional chemical images of different components of a sample using the sum filter (integral) over the marker Raman bands. In this study, chemical Raman maps of the lignified cells were generated by integrating the marker Raman peaks of lignin, cellulose and pectin over certain wavenumber ranges (Fig. 6). Considering a good signal-to-noise ratio for spectral range of 1550–1700 cm^−1^, this region is used to track lignin amounts and distribution of plant cells [57, 65]. As shown in Fig. 6a, the lignified cell was observed having strong Raman intensity and thus has high lignin concentrations. On the contrary, the surrounding parenchyma cell walls showed no Raman intensity of lignin, indicating negligible lignin concentration of the parenchyma cells compared with the lignified cells. Moreover, spatial heterogeneity of lignin concentration within the lignified cells is marked by the intensity scale in Fig. 6a. The pixels within the outer boundary of the lignified cell are red, indicating high lignin concentrations in this region.

Spatial distribution of cellulose in the lignified cells was visualized by integrating the region of 1361–1395 cm^−1^, because the Raman band centered at 1383 cm^−1^ was attributed to cellulose and shows non-orientation sensitivity [45]. As shown in Fig. 6b, the cellulose was observed saturated the whole lignified cell. On the other hand, the pectin distribution was generated by integrating the spectra over the range of 843–872 cm^−1^ with a typical Raman peak of pectin at 852 cm^−1^ (Fig. 6c), which was dominated by the C–O–C skeletal mode of α-anomers in pectin [66]. It was observed that only the cell corner and the middle lamella among the lignified cell and its adjacent parenchyma cells presented the Raman intensity of pectin, indicating the presence of pectin in these regions. Especially, the cell corner was observed having higher intensity and thus higher concentration of pectin than other regions. In addition to the 2D distribution maps, Additional file 1: Figure S2 presents three-dimensional Raman images of concentration distributions of lignin, cellulose and pectin in the lignified cell of loquat fruit, providing a direct-viewing of the distribution as well concentration variation over the lignified cell. Moreover, the characteristics of full spectra of the Raman hyperspectral images make it feasible to simultaneously visualize the components of lignin, cellulose and pectin in one image. Figure 6d shows the pseudo-color chemical image of the multicomponent lignified cell based on integrating the marker Raman bands characteristics of lignin, cellulose and pectin. The simultaneous visualization of the distributions of different components of the lignified cells provides an important manner to further understand the variation of the locations among different components of the lignified cells during fruit lignification.

**Fig. 7:**
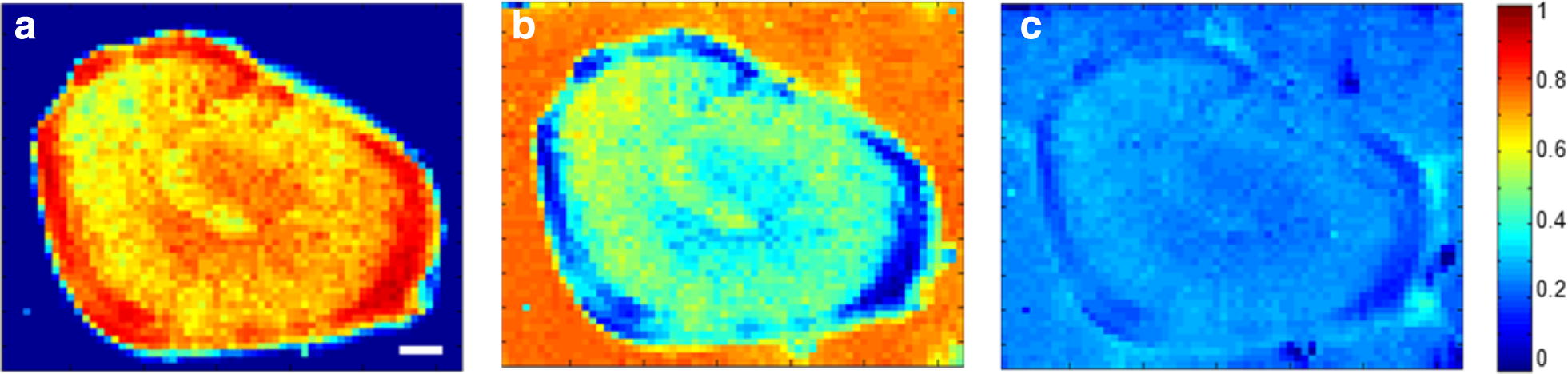
**a**–**c** Distributions of correlations between the Raman spectra of the lignified cell and those of the cell wall reference materials of lignin (**a**), cellulose (**b**), and pectin (**c**). Scale bar = 20 μm

Correlation analysis is an alternative method to analyze Raman hyperspectral images. In the present work, correlation analysis was carried out using the Raman hyperspectral image data of the lignified cells against the reference materials of cell wall, and false-colour images were produced showing the distribution of correlation coefficient responding to each pixel in the hyperspectral image. In Fig. 7a, the Raman spectra of the lignified cell showed high correlations with the lignin reference material and the outer boundary of the lignified cell exhibited higher correlations over 0.9. On the contrary, the Raman spectra of the background (parenchyma cells) showed no correlations with the Raman spectrum of the lignin reference material. As shown in Fig. 7b, the Raman spectra of the lignified cell showed weaker correlations with the spectrum of the cellulose reference material than that with the lignin reference material. The reason could attribute to that the Raman spectra of the lignified cell included several strong lignin peaks, causing a poor correlation with the spectrum of cellulose standard. Especially, the pixels with high correlation regions (mainly at the outer boundary of the lignified cell) in Fig. 7a had poorer correlation in Fig. 7b. Raman spectra from both the lignified cell and background showed scarce correlations with the pectin reference material (Fig. 7c). This was because the Raman peak of pectin in the full spectra of the lignified cell was very weak. Nevertheless, the cell corners in Fig. 7c had a similar distribution pattern of the pectin distributions in Fig. 6c. Because there are high correlations of the pixels within the lignified cell region between their Raman spectra and that of the lignin reference material, it is suggested that lignin might make a majority contribution to the Raman spectra of the lignified cell in loquat flesh. In addition, the detail of the lignin distribution inside the lignified cell was also highlighted in Fig. 7a, in which the outer boundary of the lignified cell exhibited higher correlation. Moreover, the result of the correlation analysis was in consistent with the results of lignin autofluorescence in the lignified cell in Fig. 3b.

### Lignin and cellulose variation along selected y-segment

To further visualize the concentration variations of lignin and cellulose across the lignified cell, a y-segment Raman spectra passing through the lignified cell were extracted (Fig. 6a). As shown in Additional file 1: Figure S3, Raman intensity variations of both lignin (1603 cm^−1^) and cellulose (1383 cm^−1^) were observed across the lignified cell. Raman intensity variation of lignin was more significant than that of cellulose. Stronger spectral intensity of lignin was found in the outer boundary of the lignified cell, indicating there was a higher lignin concentration in these regions. In the case of cellulose, the Raman intensity showed weak variation across the lignified cell, implying an even distribution of the cellulose.

### Distribution of functional groups of lignin in the lignified cell

**Fig. 8:**
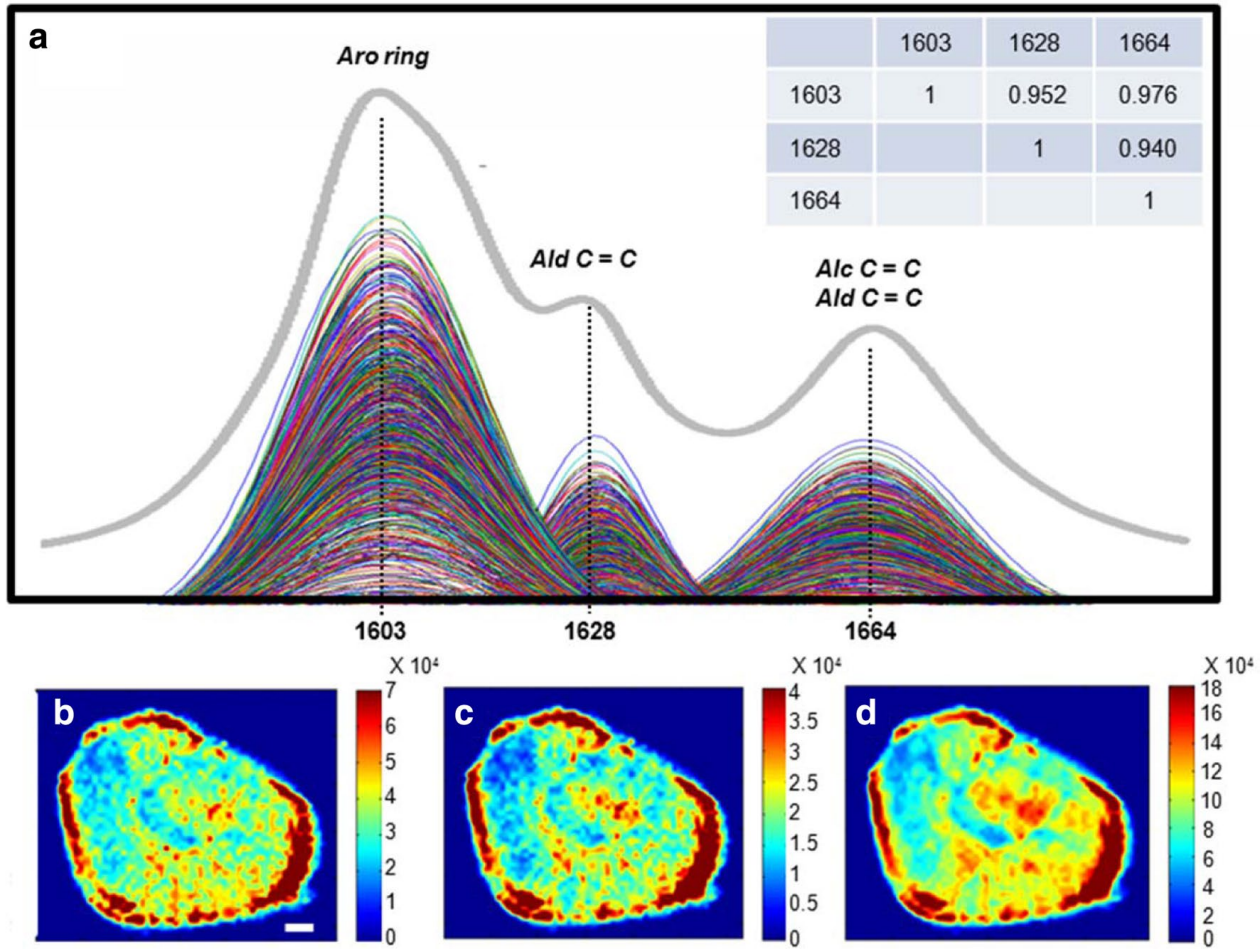
Raman images of spatial distributions of diffident functional groups of the lignin. **a** Resolved Raman peaks centered at 1603, 1628 and 1664 cm^−1^ of the overlapped fingerprint Raman bands in the spectral range of 1550–1700 cm^−1^ by multi-peaks Gaussian fitting. **b**–**d** Raman images of spatial distribution of aromatic ring, coniferaldehyde structures and confederate of coniferaldehyde and coniferyl alcohol. Scale bar = 20 μm

Lignin is a class of cross-linked racemic macromolecule that derived from oxidative coupling of a variety of monomers including aldehydes, alcohols, and other phenolic moieties [67, 68]. The three featured Raman peaks in spectral range of 1550–1700 cm^−1^ were characteristic for diffident functional groups of lignin. In specific, Raman peak at 1603 cm^−1^ was assigned to the stretch of aromatic ring in lignin; the peak at 1628 cm^−1^ was attributed to the C=C stretch in the aldehyde group; and the 1664 cm^−1^ Raman peak was attributed to the conjugated C=C and C=O modes of coniferyl alcohol and coniferaldehyde [49, 57, 58]. However, as shown in Fig. 8a, these three Raman peaks were overlapped, which made the precise visualization of the distribution of these three functional groups of lignin within the lignified cells difficult. To further scrutinize the distributions of diffident functional groups of the lignin, multi-peaks Gaussian fitting calculation was carried out to resolve the overlapped fingerprint Raman bands in the spectral range of 1550–1700 cm^−1^. As a result, the overlapped spectral peaks in the Raman spectrum of each pixel in the Raman hyperspectral image of the lignified cell were successfully resolved into three independent peaks (Fig. 8a). The fitted Gaussian peaks gave direct and exact information on the peak heights and peak areas of the sub-peaks without mutual interference. Band intensities of the fitted Gaussian peaks were observed different from those of the overlapped bands, especially an intensity reduction and shape change happened for the 1628 cm^−1^ peak. Based on the results of peak resolution, the distributions of the aromatic ring, aldehyde group as well as the confederate distribution of both aldehyde and alcohol groups of the lignin were scrutinized by evaluating the band height under fitted Gaussian peaks. Figure 8b reveled the distribution of aromatic ring of lignin of the lignified cell. The 1628 cm^−1^ Raman peak was used to construct images illustrating the distribution of coniferaldehyde structures of lignin the lignified cells (Fig. 8c). Considering that the Raman band centered at 1664 cm^−1^ attributes to coniferaldehyde and coniferyl alcohol, their confederate morphological distribution in the lignified cell is shown in Fig. 8d. There was a substantial similarity between the spatial distributions of these functional groups. As shown in Fig. 8a, it was also found that there were high correlations of band heights of pixels between peaks at 1603, 1628 and 1664 cm^−1^. Therefore, it is indicated that the contents and distributions of these three functional groups of lignin might be similar in the lignified cells.

## Discussion

A fruit is the seed-bearing structure in angiosperms formed from the ovary after flowering. Fruit has become a source of food for humans and accounted for a substantial fraction of the world’s agricultural output. Texture is an important quality attribute for fruit, as it strongly influences consumer appeal, and therefore affects market value of both fresh market and canning. Softening and lignification are two main kinds of texture variation of fruit during postharvest [15]. Lignification is the process by which monolignols units are linked together via radical coupling reactions [67]. Previous studies have provided insights into the lignification of fruit mainly in the aspects of their physicochemical and molecule biological features [10, 17, 69, 70]. Most of these physicochemical and genetic studies of the fruit lignification were based on homogenized bulk tissues with a destruction of the samples and seldom took into consideration of the multiplicity of different cell types from which the analytes are extracted, such as the metabolites, proteins or transcripts. Hence, potentially valuable spatial information will be missed and the cellular components expressed only in certain cell types or with lower abundance would be diluted.

Plant organs are composed of a multiplicity and diversity of cells and tissues that have specific structural and physiological functions. Plant lignification generates lignin polymer within the cell wall. Therefore, cellular level investigation of the deposition of lignin in fruit is of profound significance for deciphering the mechanisms underlying fruit lignification (hardening of fruit) during postharvest. Unveiling the mechanisms underlying fruit lignification during postharvest requires considering the multiplicity of different cell types in the fruit flesh and examining them in an in situ way. In the present work, Raman microspectroscopy was identified as a suitable method to investigate loquat lignification at the cell level; and this technique enabled simultaneously visualization of various polymeric compounds in a label-free manner. Spatially resolved distribution maps of lignin, cellulose and pectin revealed that lignin and cellulose saturated the whole lignified cells, pectin mainly located in the cell corner. Such simultaneous visualization of different components in one objective is important to trace the course of the formation of the lignified cells and decipher the mechanisms underlying loquat lignification. Moreover, based on the results of Raman spectral interpretation and unmixing, detailed information on lignin, including its types and functional groups distributions, was obtained dispensing with extensive sample pretreatment. It should be noted that the simultaneous visualization of different components and their functional groups is difficult to be achieved by either LM with staining or LSCM with autofluorescence.

Recently, confocal Raman imaging has shown its potential to measure molecular vibrations of cell wall and reveal the component organization within the wall of single cells in situ [45, 46, 51]. The main inherent advantages of Raman microscopy lie in the label-free nature, in situ detection, high spatial resolution (< 0.5 μm) and comprehensive information of the data. However, so far the majority of applications of confocal Raman microscopy on molecular imaging of cell walls are concentrated on investigating woody materials and energy crops, such as evaluating cell wall design and structure and function relationships [45, 47, 71–73], and monitoring the structural and chemical changes of woody materials and energy crops upon different treatments [50, 51, 74, 75]. There are only a few works that have employed this useful technique to analyze the chemical composition and spatial distribution of polysaccharides (pectins and cellulose) in the cell wall of fruits [44, 53]. Different from these studies that mainly investigated the softening mechanism of fruits, this work focused on the fruit lignification. In addition, to our best knowledge, all the above works are focusing on cell wall, whereas this study used Raman microscopic technique to investigate a certain kind of cells, called lignified cells, which contained high lignin content.

Applying confocal Raman microscopy to fruit analysis, two major challenges needed to be addressed. First, the Raman process is a weak effect and its sensitivity is relatively low. Raman signals of the fruit sample could be overwhelmed by the background fluorescence. Except for the inherent fluorescence background, some other procedures such as sample preparation (like embedding and microcutting) and setting of measurement parameters of Raman instruments would affect the background generation. In our work, microsections with high quality of the loquat flesh in the native fresh state were obtained using cryosectioning. Therefore, the lignified cells in loquat flesh exhibited a good signal-to-noise ratio of Raman spectra, especially for lignin, cellulose and pectin, making a clear and successful distribution visualization of these components in the lignified cells. Meanwhile, adaptive iteratively reweighted penalized least squares (airPLS) was used to correct baseline by subtracting the fluorescence background from the original imaging data. The fluorescence of the raw hyperspectral data were significantly reduced after the baseline correction. For the parenchyma cells, scarce Raman signal was detected. This might be because the lignin and cellulose contents in the parenchyma cells were very little, so that the chemical information of the molecular bond vibrations of the barren substances in parenchyma cell wall was very weak; and their Raman signals were overwhelmed by the fluorescence. The second challenge is to mine useful information from the overlapped Raman spectra of various molecules. In our work, multi-peaks Gaussian fitting was carried out by fitting the data points of the overlapping peaks of lignin from 1550 to 1700 cm^−1^ to a sum of N Gaussian functions. The resolved independent peaks made the possibility of analyzing aromatic ring, coniferaldehyde and coniferyl alcohol moieties of lignin in the lignified cells by evaluating the heights or areas under fitted Gaussian peaks.

In this work of investigating loquat lignification, a special kind of cells—lignified cells were found scattered in the flesh of LYQ loquat fruit. These lignified cells contained large amount of lignin, whereas the parenchymal cells were detected having little lignin according to both lignin staining and autofluorescence analysis. Label-free chemical imaging of the lignified cells by Raman microspectroscopy further confirmed there was high lignin content in these lignified cells. It is suggested that the lignified cells might be main storerooms of lignin in red-fleshed loquat fruit during lignification. Moreover, main peaks of Raman spectra of the lignified cells were assigned to lignin, cellulose, and pectin based on the spectra of their standards and using the correlation analysis. Especially, two peaks at approximately 1268 and 1335 cm^−1^ were found in the Raman spectrum of the lignified cells. These two peaks are assigned to guaiacyl (G) and syringyl (S) units of lignin, respectively [76, 77]. Therefore, it is suggested that lignin in the lignified cells of loquat fruits might be the G/S type.

## Conclusions

The methodology described in this article recommends a novel insight into the mechanisms underlying fruit lignification at the cell level using Raman microscopic imaging technique. A procedure for the simultaneous visualization of the main components of the flesh cells without labeling by high-resolution Raman microspectroscopy has been established. The research findings in this study reveal an attractive prospect about the cell characterization of loquat flesh. With Raman microscopic imaging technique, we can add a microscopic level to cell compositions, essential for a detailed molecular understanding of loquat lignification. Such method can be further used to chemically monitor the textural changes during the ripening process or postharvest storage of other fruits and vegetables.

## Materials

### Plant materials and treatment

Two cultivars of loquat fruits (*Eriobotrya japonica* Lindl. cv. ‘Luoyangqing’, LYQ and Baisha, BS) were harvested from an orchard located in Luqiao, Zhejiang, China, and were transported to the laboratory on the harvest day. Fruits of uniform commercial maturity, size and color with no mechanical wounding or disease were selected for subsequent experiments.

### Stereomicroscopy in tandem with Wiesner reaction

A binocular stereomicroscope (Carl Zeiss, Oberkochen, Germany) combined with the Weisner reagent test was employed to provide an overview about the distribution of lignin on the equatorial plane of loquat flesh at the macroscopic-scale. The stain preparation and schedule are described as follows. Loquat fruits were directly cut-through at the equatorial plane into halves with a hand knife. A few drops of 1% phloroglucinol ethanol solution was poured on the equatorial plane of the loquat flesh, adding drops of concentrated HCl to cause Wiesner reaction; then, the flesh with high lignin content was visualized after the staining. Images of the lignin-stained flesh at the macroscopic-scale were acquired immediately using the binocular stereomicroscope.

### Cryosection

Cryosection was conducted to obtain the microsections of LYQ loquat flesh in the native fresh state. The cubes (5 mm × 5 mm × 5 mm) of the flesh from the equatorial region of the fruit were impregnated directly with the optimum cutting temperature (OCT) freeze medium, snap frozen in liquid nitrogen, and then were mounted onto the ‘specimen disc’ and sectioned to a thickness of about 100 μm by a microtome (Leica, Wetzlar, Germany). The temperature of the microtome chamber was kept at − 15 °C. OCT freeze medium was removed by washing the sample with water after cutting. The prepared section was carefully put on a glass slide with a drop of water, covered by a coverslip with the thickness of 0.17 mm and sealed with nail polish to avoid evaporation.

### Laser scanning confocal microscopy

Laser scanning confocal microscopy (LSCM) was used to characterize the autofluorescence of the lignin in the loquat flesh. The flesh sections were excited by a 405 nm diode laser and images were captured under a 20× objective using a Leica TCS SP5 confocal laser scanning microscope (Leica, Wetzlar, Germany).

### Confocal Raman microscopy

Raman microscopic images in the loquat flesh at the cell level were acquired by a Renishaw inVia Reflex Raman Microscopy System (Renishaw Plc., Wotton-Under-Edge, UK) equipped with a 532 nm diode laser and an air-cooled charge-coupled device. The spectrometer was fitted with two gratings [1200 mm/line (visible) and 2400 mm/line (NIR)]. The attached microscope was a Leica DMLM equipped with four objectives (100/0.75× NA, 50/0.75× NA, 20/0.40× NA, 5/0.12× NA). The images were recorded in a spatial resolution of 2 μm in both directions, *x* and *y*, and the *z* direction was fixed. The spectra were collected over the spectral range of 1800–600 cm^−1^ under the 20× objective in static mode. Exposure time and laser power were set as 1 s and 25 mW, respectively.

For better interpretation and assignment of the Raman spectral bands of the lignified cells, the following compounds were utilized: lignin (MWL, arundo donax), commercially available cellulose (powder, ca. ~ 20 μm, Sigma Aldrich) and pectin (Citrus peel, Sangon Biotech). The Raman spectra of the cellulose and pectin standards were collected in the range of 1800–600 cm^−1^ using the Renishaw inVia Reflex Raman Microscopy System with a green laser (λ = 532 nm). For the lignin standard, a Fourier transform Raman (FT-Raman) spectroscope (Bruker, Karlsruhe, Germany) was applied to determine the spectra information with the excitation wavelength at 1024 nm. The reference Raman spectra were plotted using Origin 8.1 Pro software (OriginLab, Northampton, MA, USA).

### Light microscopy in tandem with Wiesner reaction

To verify the results of the macroscopic observation of the lignin-stained loquat flesh at the microscopic level, Light microscopy in tandem with Wiesner reaction was conducted on the flesh section. It should be noted that the acquisitions of both Raman microscopic and lignin autofluorescence images are label-free and non-destructive—in other words, their imaging processes have little influence on the cells, therefore, to simultaneously compare the results of bright field image, stained image, autofluorescence image and Raman microscopic image of the same cell, the lignin autofluorescence images and Raman microscopic images were acquired before the staining procedure. After the acquisition of Raman microscopic images, the cover slips of the section samples were removed; then, the Wiesner reaction was performed on the section. Micrographs were then captured immediately using the Renishaw inVia Reflex Raman Microscopy System. The details of the Wiesner reaction were the same as described in “Lignin staining and autofluorescence analysis for loquat flesh cells at the microscopic-scale” section.

### Spectral preprocessing and multi-peaks fitting

Spectral preprocessing algorithms are mainly used to improve the spectral data extracted from Raman micro-images mathematically. The main goal of preprocessing for Raman spectra is to correct the interference from random noise and fluorescence signals. In this work, Savitzky–Golay filter was used to reduce the noise with high-frequency in the raw Raman spectra by fitting a polynomial to each successive curve segment [78], thus replacing the original values with more regular variations. It is a very useful method to effectively remove spectral noise spikes while chemical information can be kept. On the other hand, the laser-induced fluorescence may swamp the weaker Raman scattering signals in many lignocellulosic cell walls and can limit the quality of the spectra. In this work, a baseline correction method called airPLS was used to remove the fluorescence background. In the airPLS calculation, the weights of sum squares errors (SSE) between the original signals and the fitted baseline are changed iteratively, and are obtained adaptively based on the difference between the original signals and the previously fitted baseline [79]. No prior information like peak detection etc. and user intervention are required for the airPLS calculation. In addition, many Raman bands are usually observed overlapped in the spectra of measured samples and thus limiting the resolution of the molecular information. In this work, multi-peaks Gaussian fitting was carried out by fitting the data points with multiple overlapping peaks to a sum of N Gaussian functions.

## Additional file

**Additional file 1: Figure S1.** A to F, Repetitions of bright field, fluorescence and lignin staining microscopic analysis of the loquat flesh. Scale bar = 20 μm. **Figure S2.** A to C, Three-dimensional spatial concentration distribution of lignin, cellulose and pectin in the lignified cell of loquat fruit. Scale bar = 10 μm. **Figure S3.** Baseline correction using adaptive iteratively reweighted penalized least squares (airPLS). A, Original Raman spectra; B, Raman spectra pre-processed by airPLS. **Figure S4.** Raman intensity variations of lignin (1603 cm^−1^, black line) and cellulose (1383 cm^−1^, red line) along selected y-segment. The y-sampling was conducted in 1-μm steps.

## Abbreviations

BS: cv. Baisha
LM: light microscopy
LSCM: laser scanning confocal microscopy
LYQ: cv. Luoyangqing
