# Supplementary material for: Label-free visualization of fruit lignification: Raman molecular imaging of loquat lignified cells
Source: Plant Methods. 2018 Jul 13;14:58. doi: 10.1186/s13007-018-0328-1 (PMC6043974; doi:10.1186/s13007-018-0328-1)
Supplement: Supplementary file 1 — Additional file 1: Figure S1. A to F, Repetitions of bright field, fluorescence and lignin staining microscopic analysis of the loquat flesh. Scale bar = 20 μm. Figure S2. A to C, Three-dimensional spatial concentration distribution of lignin, cellulose and pectin in the lignified cell of loquat fruit. Scale bar = 10 μm. Figure S3. Baseline correction using adaptive iteratively reweighted penalized least squares (airPLS). A, Original Raman spectra; B, Raman spectra pre-processed by airPLS. Figure S4. Raman intensity variations of lignin (1603 cm−1, black line) and cellulose (1383 cm−1, red line) along selected y-segment. The y-sampling was conducted in 1-μm steps. [file 13007_2018_328_MOESM1_ESM.pdf]

Supplemental figure 1

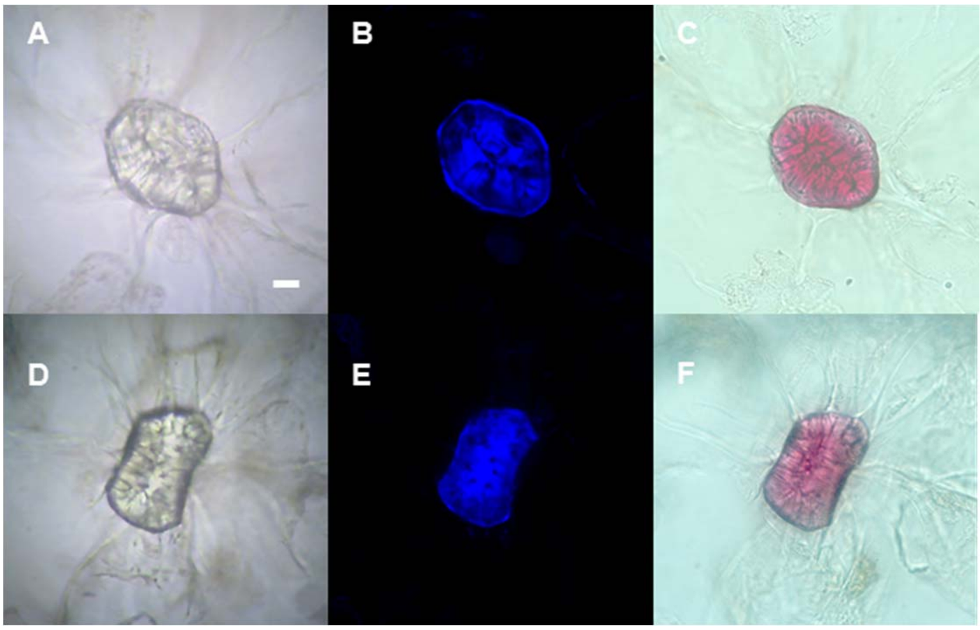

Supplemental Figure 1. A to F, Repetitions of bright field, fluorescence and lignin staining microscopic analysis of the loquat flesh. Scale bar = 20 μm.

Supplemental figure 2

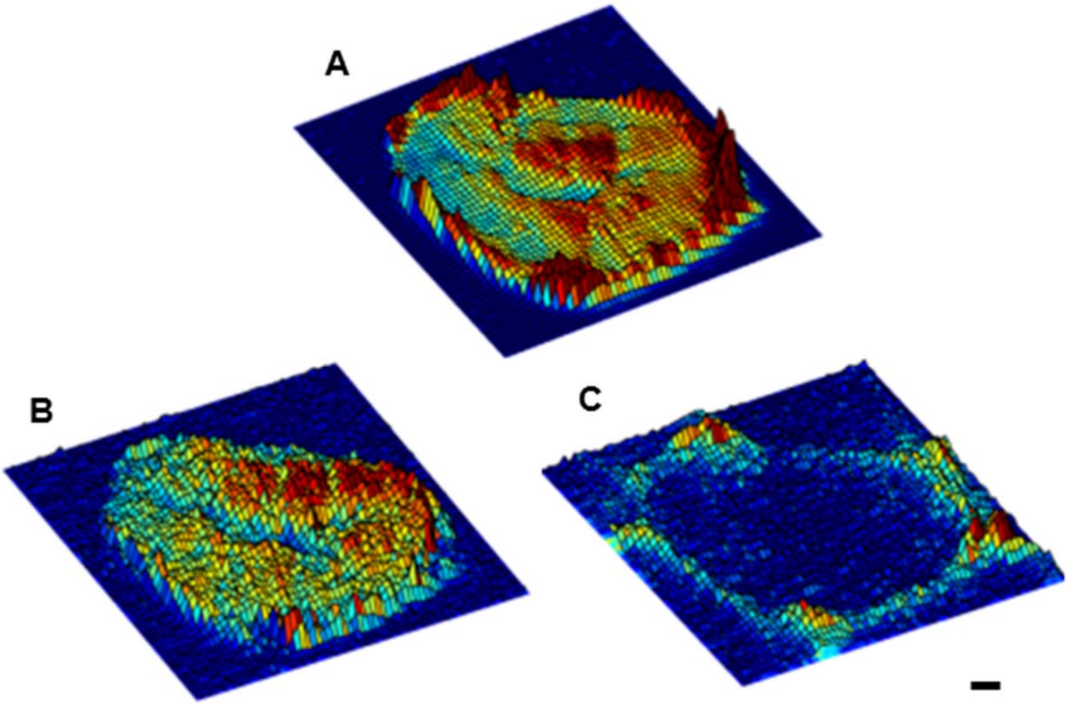

Supplemental figure 2. A to C, Three-dimensional spatial concentration distribution of lignin, cellulose and pectin in the lignified cell of loquat fruit. Scale bar = 10 μm.

Supplemental figure 3

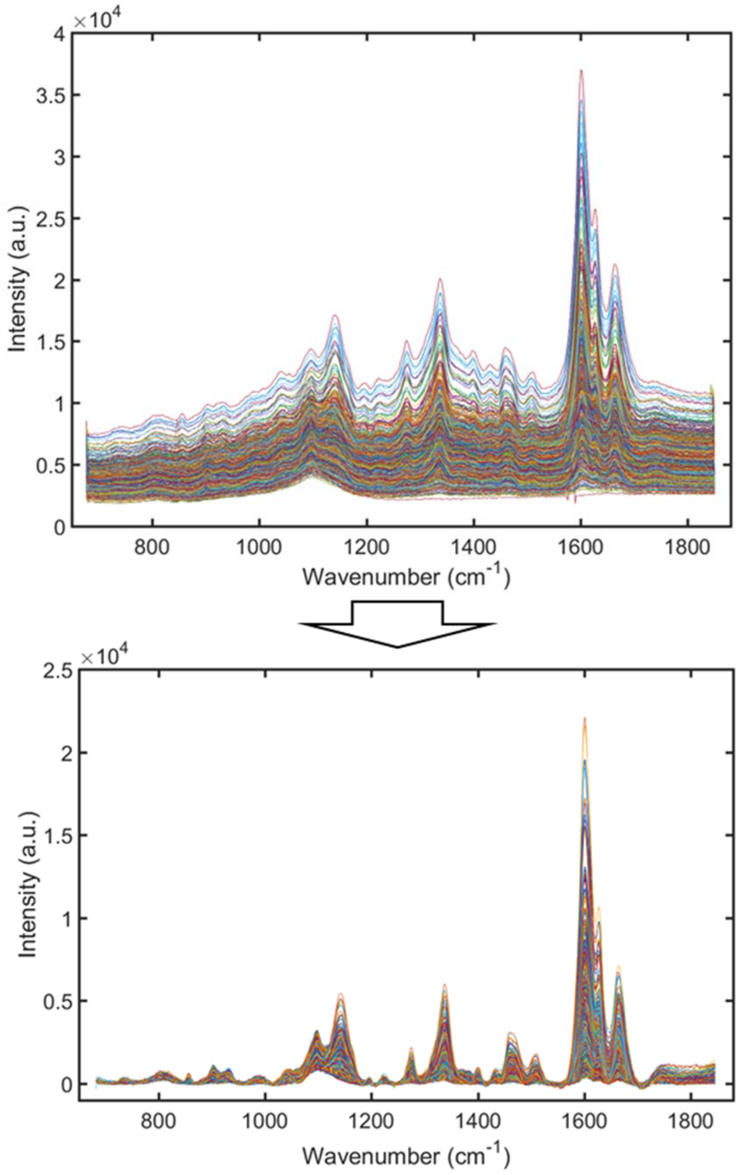

Supplemental figure 3. Baseline correction using adaptive iteratively reweighted penalized least squares (airPLS). A, Original Raman spectra; B, Raman spectra pre-processed by airPLS.

Supplemental figure 4

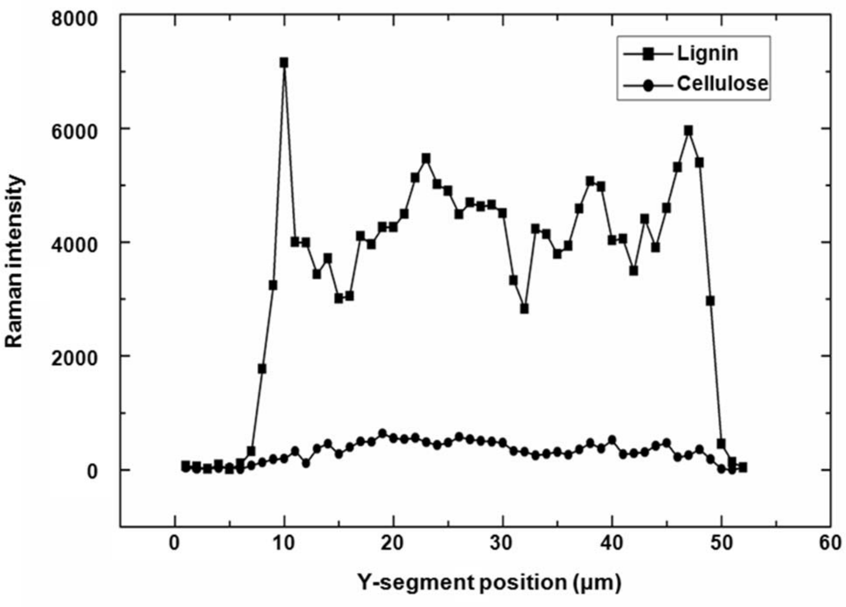

Supplemental figure 4. Raman intensity variations of lignin (1603 cm^{-1}, black line) and cellulose (1383 cm^{-1}, red line) along selected y-segment. The y-sampling was conducted in 1-μm steps.
